# Body weight, behaviours of concern, and social contact in adults and adolescents with Prader-Willi syndrome in full-time care services: Findings from pooled international archival data

**DOI:** 10.1186/s13023-024-03035-x

**Published:** 2024-02-07

**Authors:** Brian M. Hughes, Anthony Holland, Norbert Hödebeck-Stuntebeck, Lynn Garrick, Anthony P. Goldstone, Mark Lister, Craig Moore, Marguerite Hughes

**Affiliations:** 1 School of Psychology, University of Galway, Galway, Ireland; 2 Cambridge Intellectual and Developmental Disabilities Research Group, Department of Psychiatry, University of Cambridge, Cambridge, UK; 3 PWS InterNational, Bad Oeynhausen, Germany; 4 AME Community Services, Buffalo, MN USA; 5 PsychoNeuroEndocrinology Research Group, Division of Psychiatry, Department of Brain Sciences, Faculty of Medicine, Department of Endocrinology, Imperial College London, Imperial College Healthcare NHS Trust, Hammersmith Hospital, London, W12 0NN UK; 6 The Arc of Alachua County, Gainesville, FL USA; 7 Interaction Disability Services, Bella Vista, NSW Australia; 8 International Prader-Willi Syndrome Organisation, Galway, Ireland

**Keywords:** Behaviours of concern, Body weight, Prader-Willi syndrome, Specialist care, Social contact

## Abstract

**Background:**

Prader-Willi syndrome (PWS) is a complex genetic neurodevelopmental condition characterised by a range of debilitating and lifelong symptoms. The many physical and behavioural challenges that arise with adults with PWS often necessitate full-time (i.e., 24-hour) professional care support. However, despite the fact that many clinicians regard full-time PWS-specific care to represent best practice, relatively few studies have directly examined the benefits of such services. The purpose of this paper is to use archival data to investigate the impact of full-time care services on people with PWS, and to assemble a large statistical dataset on which robust analyses of improvements in weight, BMI, and behavioural outcomes can be based.

**Methods:**

Information collated by the International PWS Organisation (IPWSO), an international non-profit membership organisation supporting national PWS associations around the world, was combined into a single anonymised dataset for statistical analysis. Data were supplied by service-providers from several countries who provide full-time support to people with PWS. The dataset included details on the specific services provided, basic demographic information on service recipients, including weight, body mass index (BMI), and observational records relating to behaviours of concern (BOC; consisting of temper outbursts, skin-picking, egocentrism, inflexibility, and striving for dominance).

**Results:**

A total of 193 people with PWS (ranging in age from < 10 yrs to > 50 yrs; 93% of whom were > 18 yrs), residing in 11 services across 6 countries, were represented in the dataset. On average, people with PWS showed significant reductions in weight and BMI after joining a full-time care service, with improvements within one year of entering, which were cumulative over time and independent of age or initial weight at entry. Similar cumulative improvements over time were seen for BOC within one year and were unrelated to age or severity of BOC at entry. The degree to which services are specialised for residents with PWS appeared to confer particular benefits, with people living in PWS-exclusive services showing the greatest improvements in weight, BMI, and BOC. Reductions in BOC were associated with greater, rather than less, social contact, suggesting that these improvements were not achieved at the expense of broader freedoms, such as the opportunity to meet with families and friends.

**Conclusions:**

We conclude that full-time care services have a high likelihood of enhancing the lives of people with PWS within one year with long-lasting benefits, especially if those services are exclusive and specialised around the particular needs of PWS.

## Introduction

Prader-Willi syndrome (PWS) is a complex genetic neurodevelopmental condition characterised by a range of debilitating and lifelong symptoms. PWS arises from the absence of paternal copies of genes that are maternally imprinted, for one of three reasons: a deletion on chromosome 15 at the locus q11-13, the presence of a maternal chromosome 15 uniparental disomy, or an imprinting centre defect at the chromosomal locus 15q11-13 [1]. Usually it is classified as a rare condition, given a population prevalence that varies from 1:50,000 to 1:30,000 [2]. Because of this rarity, there can be limited familiarity or understanding of PWS among those working in healthcare and social services systems. Support services often vary very widely between countries and from region to region, an inconsistency that is likely to undermine not only the welfare of people with PWS and that of their families, but also the quality of medical knowledge regarding the types of support services from which people with PWS will most benefit.

The symptoms of PWS include mild or moderate intellectual disability, low muscle tone (hypotonia), hormone deficiencies (especially growth hormone and sex hormones) with incomplete sexual development, delays in language and motor development, poor social awareness, and anxiety [3], as well as diminished affective perspective-taking ability [4]. People with PWS also exhibit an array of self-limiting behaviours that cause great difficulty to themselves and those around them, including temper outbursts, obsessional hoarding, striving for dominance, egocentrism, inflexibility, and skin-picking [5]. A feature that is particularly characteristic of PWS is a lack of satiety or appetite control that produces an overwhelming drive to eat (hyperphagia), resulting in excessive calorie intake and a risk of life-threatening obesity.

Hyperphagia, which arises in virtually all people with PWS from early childhood onwards, is believed to be associated with impairments in neural mechanisms mediating the satiety cascade, which catastrophically disrupt feelings of fullness [6]. Hyperphagia causes significant difficulties, rendering a person with PWS limited in their ability to restrict their own eating and so unable to manage their own food intake [7]. The risks posed by uncontrolled access to food are therefore life-threatening. Based on data from 500 persons with PWS who had died, Bellis et al. [8] demonstrated that hyperphagia was either directly or indirectly implicated in 77% of fatalities. In addition to obesity-related mortality (such as that arising from type 2 diabetes mellitus, cardio-respiratory failure and arrest, pulmonary embolus, and infections), hyperphagia can also prove fatal due to its role in promoting choking, gastric rupture, or toxin ingestion. As such, improving life-expectancy for people with PWS largely depends on the successful management, through improved support, of the immediate and long-term obesity- and non-obesity-related risks posed by hyperphagia [8]. Without the benefits of such support, a person with PWS will likely need independent assistance for every single meal and 24-hour food supervision over their lifetime.

Such meticulous control of the food environment, such as locked food access and continuous supervision, amounts to the only ‘treatment’ or ‘management’ currently available for hyperphagia [9]. When describing their own aspirations, people with PWS typically identify control of the food environment as being fundamental to the type of care they wish to receive [10]. Families attempting to independently meet this considerable care demand typically experience significant levels of stress and greatly diminished quality of life [11–12]. Advocacy groups report that many families continue to be the main source of support because they believe that only full-time or residential care settings can provide the supports needed to ensure their family member’s welfare [13]. As a result, in the absence of alternatives, people with PWS often continue to live in family settings well into adulthood, with more than 40% of those in the United States aged 40 years and above continuing to do so [14].

Nonetheless, because of the many physical and behavioural challenges that arise (from hyperphagia as well as from other PWS-specific cognitive and social phenotypes), people with PWS frequently require a full-time formal care service – in which an individual receives 24-hour support from professional care staff – often provided in a residential or supported-living setting. In such formal care contexts, a tension that arises is whether the required level of food control arrangements can be appropriately provided in mixed care settings, or whether hyperphagia-specific arrangements are needed to protect the health and well-being of people with PWS. Clinicians who specialise in PWS widely regard continuous supervision and locked kitchens to be two important elements of full-time PWS-specific care that epitomise best practice (along with other environmental and interpersonal elements, as well as essential staff skills, attributes, and training; see [15]). However, despite this, there have been relatively few studies to directly examine the benefits that such services provide. In general terms, this is most likely due to the comparative rarity of PWS and the consequent difficulty in assembling datasets that are large enough to account for the many complexities inherent in the condition.

In one early study, Mullins and Vogl-Maier [16] examined weight maintenance among a group of nine children with PWS who attended group rehabilitation in a residential setting. They reported that across a three-year period, the children successfully maintained a consistent body weight, in contrast to the rapid weight gain commonly seen in ‘untreated’ children with PWS. However, by necessity, the study was observational in nature, and lacked a sufficient sample size to allow for statistical analysis of data. Of the nine children included in the study, one dropped out partway through. The residential nature of the service described was quite parsimonious, comprising just 26 days per year, implying that the observed effects on weight maintenance may have only partially resulted from on-site rehabilitation activities. Nonetheless, this early study helped to illustrate the potential improvements in the lives of people with PWS that could be offered by structured residential programmes.

Hirsch et al. [17] attempted to compare long-term changes in body mass index (BMI) in adults with PWS living in care hostels with that of adults with PWS living with families at home. They reported that individuals living in hostels had lower BMI compared to individuals living in family homes. However, despite the use of a meticulous study design, the evidence supporting this conclusion was limited by the restricted nature of the study sample. While data had originally been gathered from an initial group of 34 individuals with PWS in residential care, the need for age-matching meant that only 17 such individuals were included in the statistical analysis. Moreover, the two comparison groups differed in gender, with a majority of hostel residents being male but a majority of home residents being female. Finally, there was considerable variability in the lengths of time that the individuals in the hostel group had spent in residential care, which was not factored into the statistical analyses. The analyses were largely based on two-point univariate comparisons (e.g., *t*-tests and repeated-measures analysis of variance), as the small sample size precluded the use of multivariate tests.

In an effort to garner a larger dataset, Bedard et al. [18] reviewed archival information relating to 45 adults with PWS who received a comprehensive treatment package, focusing on management of weight and behaviour, while residing in a care service in Alachua County, Florida, United States. Their analyses showed general reductions in weight, food stealing, tantrum behaviour, skin-picking, and self-injury, suggesting that the services provided had beneficial effects. However, the sample size was again generally too small to permit detailed statistical analysis, especially within subgroups of interest (such as different age ranges). In addition, the researchers restricted their analyses to individuals with PWS who had lived in full-time care for a minimum of six years prior to baseline assessment. While this inclusion criterion helped to standardise the dataset, it necessarily meant that improvements in weight or behaviour occurring during the first six years of residential care were not covered by the study.

Cross-sectional analysis of data from adults under the care of a PWS national referral hospital in Rotterdam, Netherlands [19], revealed that people living in a PWS-specialist home (*n* = 23) had lower BMI compared to those who lived in a non-PWS specialist home (*n* = 61) (median [interquartile range] = 27 [22–30] vs. 30 [27–40]). They also exhibited a lower prevalence of hypertension (0% vs. 29%) and a lower prevalence of Type 2 diabetes mellitus (9% vs. 22%), although the latter difference was not statistically significant. However, interpretation of these results was complicated by the fact that the people with PWS who lived in specialist homes were younger than those who lived in non-specialist homes (26 [21–32] vs. 36 [28–50] years).

These studies illustrate the challenges facing researchers who attempt to examine the impact of residential care in conditions such as PWS. However, despite the ingenuity shown by the researchers who designed these informative studies, the restricted nature of the various datasets and research contexts greatly reduces the strength of each study’s conclusions. As a result, the existing literature on outcomes resulting from full-time specialist care in PWS is severely limited. The purpose of this paper is to expand the practice of using archival data to shed light on the impact of full-time specialist care services on people with PWS, and to assemble a large statistical dataset on which robust analyses of improvements in weight, BMI, and behavioural outcomes can be based. As well as increasing the statistical power of analyses, this paper draws data from a wide range of service settings, including from different countries, and as such aims to produce findings that are not bound to any single place, time, or cultural context. By both expanding and enriching the dataset used for research, we aim to provide usable insights as to the utility of full-time specialist care as a means to improve the lives of people with this condition, and to assist people with PWS, and their families, in identifying the types of services that they wish to access. The hypotheses were as follows: (i) that people with PWS entering care settings gain benefit in terms of reductions in weight/BMI and improvements in behaviours of concern (BOC), (ii) that these beneficial outcomes are greater in PWS-exclusive rather than in mixed care settings, and (iii) that improvements in BOC are not associated with restricted social contact.

## Methodology

### Call for archival data

The analyses presented here are based on data collated by the International Prader-Willi Syndrome Organization (IPWSO). IPWSO was founded in 1991 as an international non-profit membership organisation supporting national PWS associations around the world, as well as assisting people with PWS, their families, and the professionals who work with them. As an umbrella organisation, IPWSO works extensively with a wide range of national PWS associations, as well as with practitioners, parent groups, and other patient advocates in territories where no formal associations have been established. As a result, IPWSO has active contacts in well over 100 countries, and is a truly world-wide parent-led support organisation [20].

As part of its ongoing information-gathering activities, in July 2021, IPWSO issued a call to care providers for archival data on PWS services, via its member organisations, subcommittees, and other various stakeholders. Interested parties were invited to conduct historical file reviews and to collaborate in pooling relevant data, drawn from their records, that could at scale be used to examine the lives, well-being, and outcomes of individuals with PWS who enter full-time care services. The information submitted was combined into a single generic dataset.

Service providers were invited to supply archival data that they already had on file and which they were willing and permitted to share, relating to individuals with PWS to whom they were providing full-time care services. Providers submitted fully anonymised information on a standardised spreadsheet to allow data to be pooled by IPWSO into a single generic dataset. Types of data to be included covered basic demographic information, basic information on individuals’ weight and/or BMI, frequency of their contact with family and friends, as well as details about the different services provided, such as the numbers and types of staff who provided care to each individual.

Providers were also asked if they had any observational records relating to individuals’ BOC at entry, after one year, and currently. The specific BOC identified were temper outbursts, skin-picking, egocentrism, inflexibility, and striving for dominance, each of which is commonly seen in PWS. For each BOC, providers were asked to provide a rating reflecting the severity of the specific BOC at the relevant time point (see Table 1). For the purposes of pooling data, ratings of BOC severity were transformed to a scale of 1 (minimum) to 4 (maximum), and an overall BOC score was computed as the average of ratings recorded for each individual.

**Table 1:** Questions relating to behaviours of concern (BOC)

Time point	Questions
At entry	*Did the person experience problems with* *temper outbursts* *(e.g. ‘meltdowns’) when they entered your service?* *Did the person experience problems with* *skin-picking* *when they entered your service?* *Did the person have problems with* *egocentrism* *when they entered your service? (i.e., focusing on themselves and unable to see other people’s point of view)* *Did the person have problems with* *inflexibility* *when they entered your service? (e.g., difficulty with changes in routine)* *Did the person have problems with* *striving for dominance* *when they entered your service? (e.g., wanting to have their own way/insisting on being in control)*
After one year	*Did the person experience problems with* *temper outbursts* *(e.g. ‘meltdowns’) ONE YEAR AFTER they entered your service?* *Did the person experience problems with* *skin-picking* *ONE YEAR AFTER they entered your service?* *Did the person have problems with* *egocentrism* *ONE YEAR AFTER they entered your service? (i.e., focusing on themselves and unable to see other people’s point of view)* *Did the person have problems with* *inflexibility* *ONE YEAR AFTER they entered your service? (e.g., difficulty with changes in routine)* *Did the person have problems with* *striving for dominance* *ONE YEAR AFTER they entered your service? (e.g., wanting to have their own way/insisting on being in control)*
Now	*Does the person experience problems with* *temper outbursts* *(e.g. ‘meltdowns’) NOW?* *Does the person experience problems with* *skin-picking* *NOW?* *Does the person have problems with* *egocentrism* *NOW? (i.e., focusing on themselves and unable to see other people’s point of view)* *Does the person have problems with* *inflexibility* *NOW? (e.g., difficulty with changes in routine)* *Does the person have problems with* *striving for dominance* *NOW? (e.g., wanting to have their own way/insisting on being in control)*

Note: Response options, as rated by staff, for all questions were as follows: *Severe problems with…* (scored in the analyses as ‘4’)/*Moderate problems with…* (‘3’)/*Minor problems with…* (‘2’)/*No problems with…* (‘1’)

Providers were asked if they had any record of frequency of social contact. They were invited to provide a rating of “approximately how much contact” the individual currently has with (a) their family and (b) their friends, based on scales ranging from 0 (“no contact”) to 4 (“daily contact”). Providers were advised that such social contact could be in-person (such as visits) or virtual (e.g., telephone or video calls) and that ‘friends’ in this context should refer to persons other than professional caregivers (and as such, could include other service recipients or cohabitants in a service setting as well as social contacts from work or the wider community). For the purposes of analysis, ratings relating to friend and family contact were averaged to produce an overall index of current social contact.

As all the data to be supplied were archival, providers were free to incorporate whatever additional information they felt would be relevant; accordingly, the resulting dataset reflected the emergent nature of the information gathering process and was not constrained by detailed prescribed inclusion criteria.

### Statistical analysis

All measures of central tendency are presented as mean ± SD. Data were assessed for normal distribution using visual inspection. This approach was adopted for multiple reasons, including the fact that statistical methods for evaluating normality can be oversensitive (or under-sensitive) depending on sample characteristics, which is why visual inspection of distributions remains an important technique [21]. In addition, while normal distributions are an assumption of parametric tests, they are not an essential requirement: such tests are famously robust against violations of the normality assumption, and perform well especially when variables are skewed in common directions [22]. Some variables, such as weight and BMI, can be expected to vary substantially when there is a broad age range of persons in a dataset. In our dataset, variables were often skewed slightly positively (i.e., with a small subset of outliers with high values), rendering the variables non-normal but nonetheless amenable to parametric tests. This being said, we double-checked all parametric analyses reported below by conducting equivalent non-parametric statistical tests to confirm the overall pattern of statistical effects.

Aided by such double-checking, we employed parametric statistical tests where visual inspection suggested that variables were adequately distributed, and non-parametric statistical tests where visual inspection suggested that variables were excessively skewed. Comparisons of outcome variables between time-points were performed using paired Student *t*-tests. Bivariate associations were computed as Pearson correlations for the majority of variables, except when we observed that data were highly skewed, in which cases Spearman correlations were used instead. Multivariate associations were computed using multiple linear regression. When comparing changes in outcomes over time, mixed factorial Analysis of Covariance (ANCOVA) was used to control for potential confounding variables; similarly, partial correlation was used to adjust for potential confounding variables when considering associations. All analyses were conducted using SPSS version 26.

## Results

### Dataset

Data were supplied by 11 full-time care services from across six countries, namely, Denmark, Germany, Ireland, Switzerland, the United Kingdom, and the United States. At least partial data were supplied for services provided to a total of 193 individuals with PWS, who were in receipt of the relevant services for an average of 10.26 yrs (ranging from < 5 to > 40 yrs). The average age at which these individuals first entered the services that they were now receiving was 26.67 yrs (ranging from < 10 yrs to > 50 yrs). The majority of individuals (93%; *n* = 180) were more than 18 years old at entry. Detailed information on age and on length of residence is presented in Table 2.

**Table 2:** Age and time in service of individuals in overall dataset (*N* = 193)

	M ± SD	Range
Current age (yrs)	37.92 ± 13.30	14–87
Age at entry (yrs)	27.66 ± 9.84	9–65
Time spent in service (yrs)	10.26 ± 8.15	< 1–43

Given the archival nature of the dataset, only partial data relating to key variables of interest were available for most individuals. The overall scale of the dataset ensured that sufficient data were available to test several important hypotheses as many of the analyses presented below were based on subsets of participants drawn from the overall sample of *N* = 193, with differing sub-sample sizes specified for each separate analysis.

### Changes in body weight and BMI

Summary data on body weight and BMI at entry, after one year, and currently are presented in Table 3, clustered by paired samples (i.e., subsets of individuals for whom data were available at two time-points) and isolated to individuals aged 18 or over at entry. Significant reductions over time were recorded across all of these paired samples for both body weight and BMI (see Table 3).

**Table 3:** Body weight and BMI at entry, after one year and currently, for persons aged 18 or over at entry

	n	M	SD	t (df)	*p*
Body weight (kg)					
*Paired sample #1*					
At entry	145	101.89	40.32	10.76 (144)	< 0.001
Currently	145	71.74	17.82		
*Paired sample #2*					
At entry	36	80.75	29.92	4.07 (35)	< 0.001
After one year	36	68.32	16.02		
BMI (kg/m^2^)					
*Paired sample #3*					
At entry	141	41.45	16.26	19.65 (140)	< 0.001
Currently	141	29.14	6.96		
*Paired sample #4*					
At entry	34	31.47	7.98	5.32 (33)	< 0.001
After one year	34	27.40	5.34		

Note: *n* = number of cases in each paired sample; *M* = mean; *SD* = standard deviation; *t(df)* = test statistic produced by *t*-test for the given degrees of freedom; *p* = probability value used for statistical significance testing

Changes of body weight may be contingent on an individual’s age, which may be pertinent here given the wide range of ages at which individuals entered services. However, across the dataset for individuals who were aged 18 or over at entry, age at entry was not significantly correlated with weight at entry (*n* = 164, *r* =–.153, *p =*.051), overall weight loss to date (*n* = 119, *r* =–.130, *p =*.119), or weight loss after one year (*n* = 36, *r* =–.200, *p =*.241).

Mixed factorial ANCOVA was used to examine whether the changes in body weight and BMI were affected by age, by incorporating age at entry as a covariate. Both the observed overall reductions in body weight (*F*(1,143) = 24.78, *p* <.001) and the observed reductions in body weight after one year (*F*(1,34) = 6.79, *p* =.013) remained statistically significant after controlling for individuals’ ages at entry, confirming that statistical changes in weight in this dataset were independent of age at entry. Similarly, overall reductions in BMI (*F*(1,139) = 25.67, *p* <.001) and reductions in BMI after one year (*F*(1,32) = 11.62, *p* =.002) also remained significant after controlling for age.

**Fig. 1:**
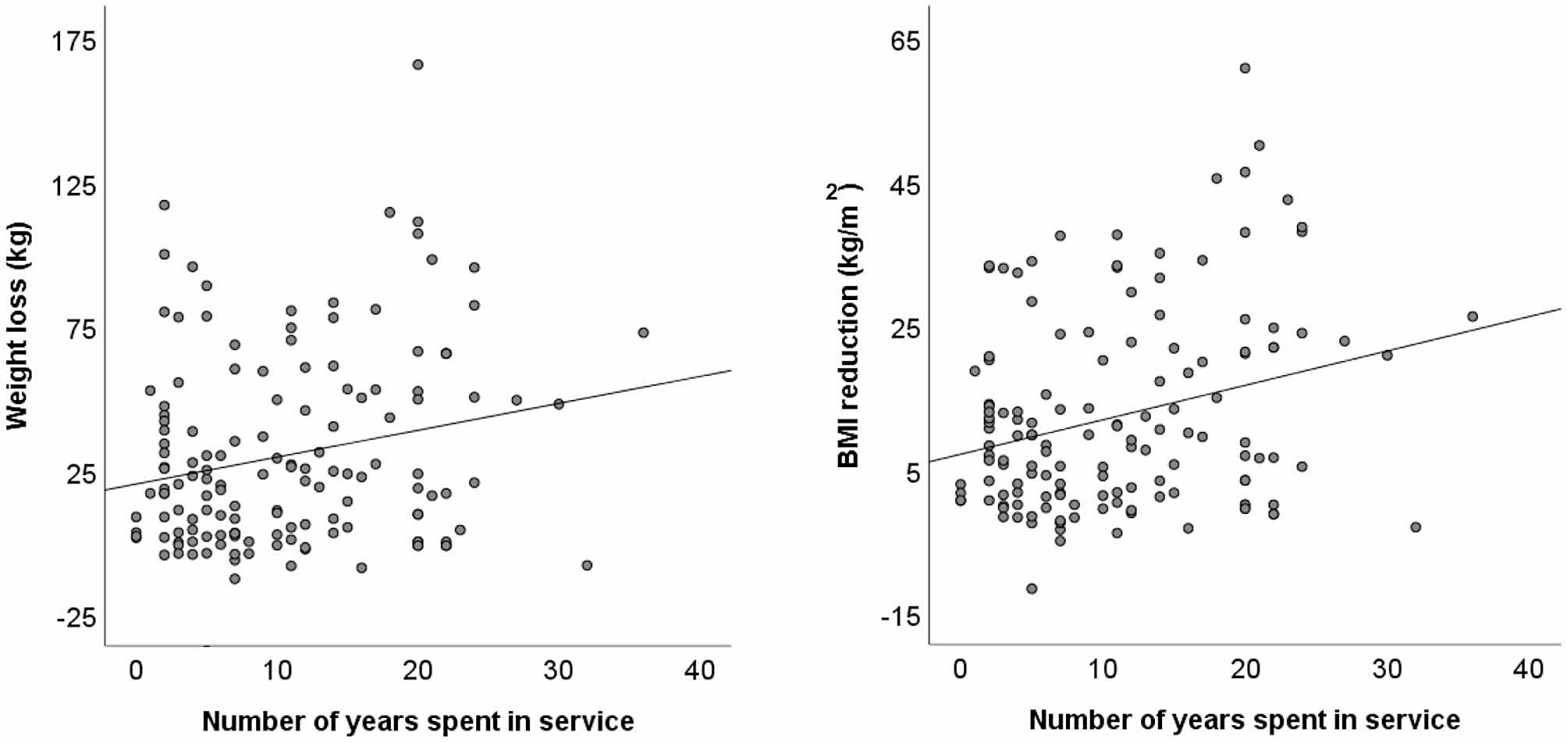
Relationship between changes in weight and BMI with years spent in service Note: Scattergrams show overall reductions in weight (left; *n* = 145) and BMI (right; *n* = 141) since entering a service as a function of number of years spent in that service. Reductions in both weight and BMI increased significantly with the passing of time

Given the wide range of timeframes within which individuals were in services (min–max 1–43 yrs), we also examined whether number of years spent in a service was associated with observed overall reductions in weight and BMI. With regard to body weight (*n* = 145), number of years spent in a service was significantly correlated with overall reduction in weight (*r* = + 0.216, *p* =.009; see Fig. 1). This effect remained significant when controlling for age using partial correlation (*r*_p_ = + 0.228, *p* =.006) and when excluding one particular individual whose extreme weight loss (in excess of 150 kg after around 20 years) constituted an outlier (*r*_p_ = + 0.207, *p* =.013). Based on conventional thresholds, correlation coefficients around 0.20 correspond to ‘small’ to ‘medium’ effect sizes [23]. Incorporating number of years in a service as a second covariate in the ANCOVA for weight loss revealed that length of time in a service was, in fact, the primary independent variable predicting reductions in weight (*F*(1,140) = 11.59, *p* =.001), with the index of effect size indicating a ‘medium’ to ‘large’ effect (partial η^2^ = 0.076) (Table 4).

**Table 4:** Factors influencing weight and BMI change over time

Predictor	Sum of squares	df	Mean square	F	*p*	Partial η^2^
Weight change	6.75	1	6.75	0.01	0.904	0.000
Weight change × Age	975.69	1	975.69	2.09	0.150	0.015
Weight change × Time in service	5409.36	1	5409.36	11.59	0.001	0.076
Weight change × Age × Time in service	3402.93	1	3402.93	7.292	0.008	0.050
Error	65329.63	140	466.64			
BMI change	0.001	1	0.001	0.00	0.998	0.000
BMI change × Age	134.57	1	134.57	1.75	0.188	0.013
BMI change × Time in service	993.61	1	993.61	12.93	< 0.001	0.087
BMI change × Age × Time in service	536.30	1	536.30	6.98	0.009	0.049
Error	10451.68	136	76.85			

Note: Mixed factorial ANCOVA results for overall changes in weight (*n* = 144) and BMI (*n* = 140) with possible influence of age at entry and number of years in service examined as covariates; Sum of squares, *df*, Mean square, and *F* refer to ANOVA test results; *p* = probability value used for statistical significance testing; Partial η2 = effect size statistic

Similarly, number of years spent in a care setting was significantly correlated with reductions in BMI (*n* = 141; *r* =.272, *p* =.001; see Fig. 1), again after both controlling for age (*r*_p_ = + 0.284, *p* =.001) and removing the outlier (*r*_p_ = + 0.267, *p* =.002). Conventional thresholds suggest that correlations of around 0.3 represent ‘medium’ effect sizes [23]. As with body weight, ANCOVA confirmed that length of time in a service (as a second covariate to age at entry) was the primary predictor of reduced BMI (Table 4).

### Changes in behaviours of concern

**Fig. 2:**
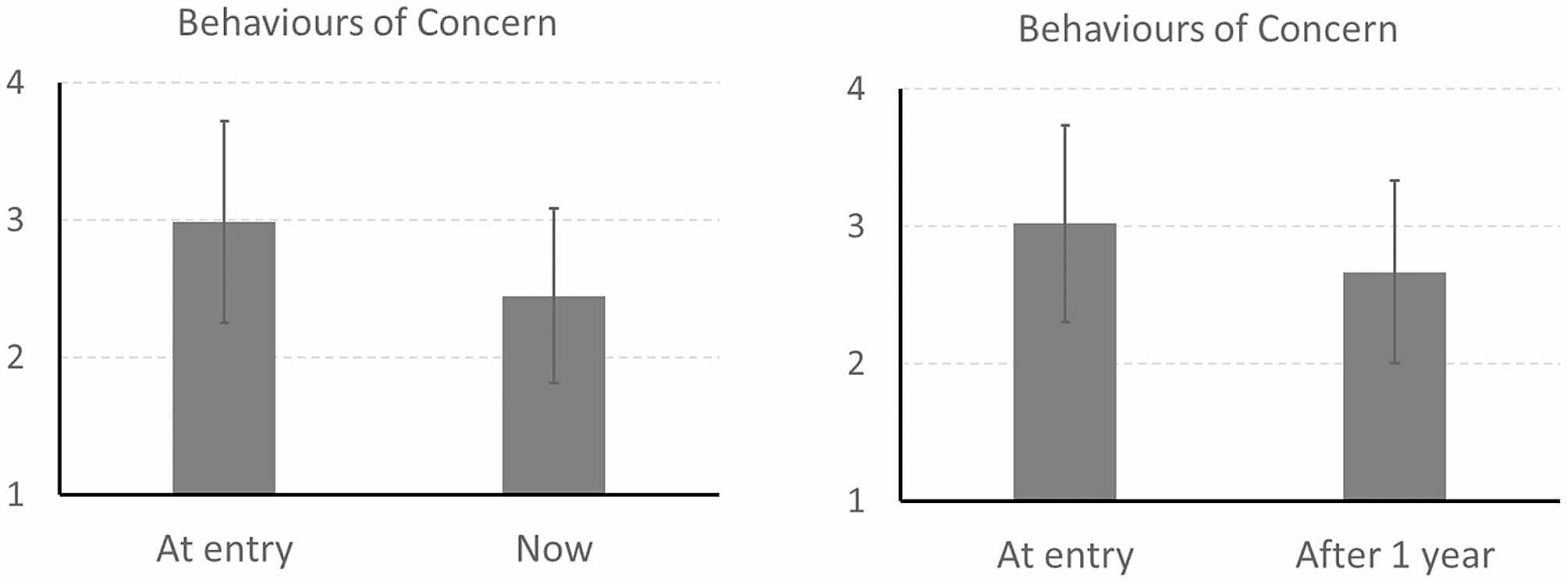
Changes in behaviours of concern over time Note: Comparisons of behaviours of concern (BOC) at entry with current BOC (left; *n* = 41) and BOC after one year (right; *n* = 39). Bars denote mean and error bars denote standard errors of the mean. Reductions within both time frames were statistically significant (*p* <.001 for current BOC; *p* =.001 for BOC after one year), including after controlling for age, and both constituted ‘large’ statistical effects (partial η^2^ = 0.279 for current BOC; partial η^2^ = 0.271 for BOC after one year)

Data on BOC *both* at entry *and* currently were available for 41 individuals, and showed significant reductions in BOC over time (see Table 5). Mixed factorial ANCOVA showed that this reduction in BOC was statistically significant even after controlling for age (see Table 6). Data on BOC both at entry and one year later were available for 39 individuals, and again showed significant reductions over time (Table 5). This reduction was also significant after controlling for age (Table 6). The overall reduction in BOC (partial η^2^ = 0.279) and the reduction in BOC after one year (partial η^2^ = 0.271) constituted very ‘large’ statistical effects (Fig. 2).

A further ANCOVA suggested that these reductions in BOC remained significant even after controlling for *both* age *and* length of time in the service (*F*(1,38) = 19.31, *p* <.001, partial η^2^ = 0.337 for current BOC; *F*(1,36) = 21.07, *p* <.001, partial η^2^ = 0.369 for BOC after one year). However, given the small sample size, such ANCOVA results involving multiple covariates should be interpreted with caution.

**Table 5:** Behaviours of concern at entry, after one year and currently

	n	M	SD	t (df)	*p*
BOC					
*Paired sample #1*					
At entry	41	2.99	0.73	5.98 (40)	< 0.001
Currently	41	2.45	0.64		
*Paired sample #2*					
At entry	39	3.02	0.72	4.96 (38)	< 0.001
After one year	39	2.67	0.66		

Note: BOC were rated from 1 (minimum) to 4 (maximum); *n* = number of cases in each paired sample; *M* = mean; *SD* = standard deviation; *t(df)* = test statistic produced by *t*-test for the given degrees of freedom; *p* = probability value used for statistical significance testing

**Table 6:** Factors influencing change in behaviours of concern over time

Predictor	Sum of squares	df	Mean square	F	*p*	Partial η^2^
BOC change overall	2.36	1	2.36	15.10	< 0.001	0.279
BOC change overall × Age	0.55	1	0.55	3.55	0.07	0.08
Error	6.09	39	0.16			
BOC change after one year	1.26	1	1.26	13.76	0.001	0.271
BOC change after one year × Age	0.38	1	0.38	4.16	0.049	0.101
Error	3.40	37	0.09			

Note: Sum of squares, *df*, Mean square, and *F* refer to ANOVA test results; *p* = probability value used for statistical significance testing; Partial η2 = effect size statistic

### Predictors of improvement

The following features of the care services received were available for 91 individuals who also had BMI recorded: (i) staff specialisation (i.e., specialisation in PWS care, defined in this case as whether care staff work exclusively with individuals with PWS *or* whether the care staff work with a variety of clients), (ii) service specialisation (i.e., based on the client population in a given setting, defined in this case as whether the individual lives only with other individuals with PWS *or* whether the individual lives with a variety of other people), (iii) food control arrangements (i.e., whether the individual has no access to food outside of mealtimes *or* whether they have such access); while 41 individuals also had data on (iv) number of cohabitants (i.e. the number of other people with whom the individual lives in the residential setting) (Table 7).

The possibility that staff specialisation, service specialisation, and/or food control arrangements might be associated with current BMI was initially examined using point-biserial correlation coefficients. Each service feature variable was coded as 0 or 1 and a series of bivariate correlations were computed between them and current BMI. All three were found to be significantly associated with current BMI at a bivariate level (*r* =.236, *p* =.025 for staff specialisation; *r* =.305, *p* =.003 for service specialisation; *r* =.274, *p* =.009 for food control arrangements). Once again, when judged against conventional thresholds [23], such correlations correspond to ‘medium’ effect sizes. Multiple regression was then used to examine all three predictors simultaneously, in order to compare their relative influence in predicting current BMI. A regression model including all three predictors together significantly predicted BMI overall (*F*(3,90) = 3.73, *p* =.014). Within this regression model, service specialisation was the only service feature to predict current BMI (β = 0.531, *p* =.054). This association was interpreted based on a post hoc *t*-test, which showed that the current BMI of individuals who live with other individuals with PWS was significantly lower (26.68 ± 5.69 kg/m^2^) than that of individuals who live in a mixed setting (32.65 ± 11.33 kg/m^2^), an average difference of 5.97 kg/m^2^ (*t*(89) = 3.02, *p* =.003).

**Table 7:** Features of care services received

Service feature	Details
Staff specialisation (*n* = 91)	*n (%)*
*Care staff work exclusively with individuals with PWS*	75 (82.4%)
*Care staff work with individuals with PWS and with other clients*	16 (17.6%)
Service specialisation (*n* = 91)	*n (%)*
*Individual lives only with others who have PWS*	77 (84.6%)
*Individual lives with a variety of other individuals*	14 (15.4%)
Food control arrangements (*n =* 91)	*n (%)*
*Individual has no access to food outside of mealtimes*	77 (84.6%)
*Individual has limited access to food outside of mealtimes*	14 (15.4%)
Number of cohabitants (*n* = 41)	*M* ± *SD (range)*
*Number of other residents the individual currently lives with*	10.66 ± 9.98 (2–29)

The possibility that staff specialisation, service specialisation, food control arrangements, and/or number of cohabitants might be associated with improvements in BOC was examined similarly, in the first instance using point-biserial correlation coefficients to compute the association of each service feature separately with changes in BOC over time. Both service specialisation (*r* =.33, *p* =.034) and number of cohabitants (*r* =.68, *p* <.001) significantly correlated with changes in BOC over time, whereas both bivariate correlations for staff specialisation (*r* =.15, *p =*.344) and food control arrangements (*r* =.28, *p* =.073) with BOC were non-significant. As above, multiple regression was then used to examine all four service features together, in order to ascertain their relative roles in predicting BOC outcomes. When the four service features were analysed together, service specialisation was no longer significantly associated with changes in BOC. However, number of cohabitants (β = 0.69, *p* <.001) remained a significant predictor of BOC improvements in the multiple regression model (*F*(4,40) = 12.68, *p* <.001).

**Fig. 3:**
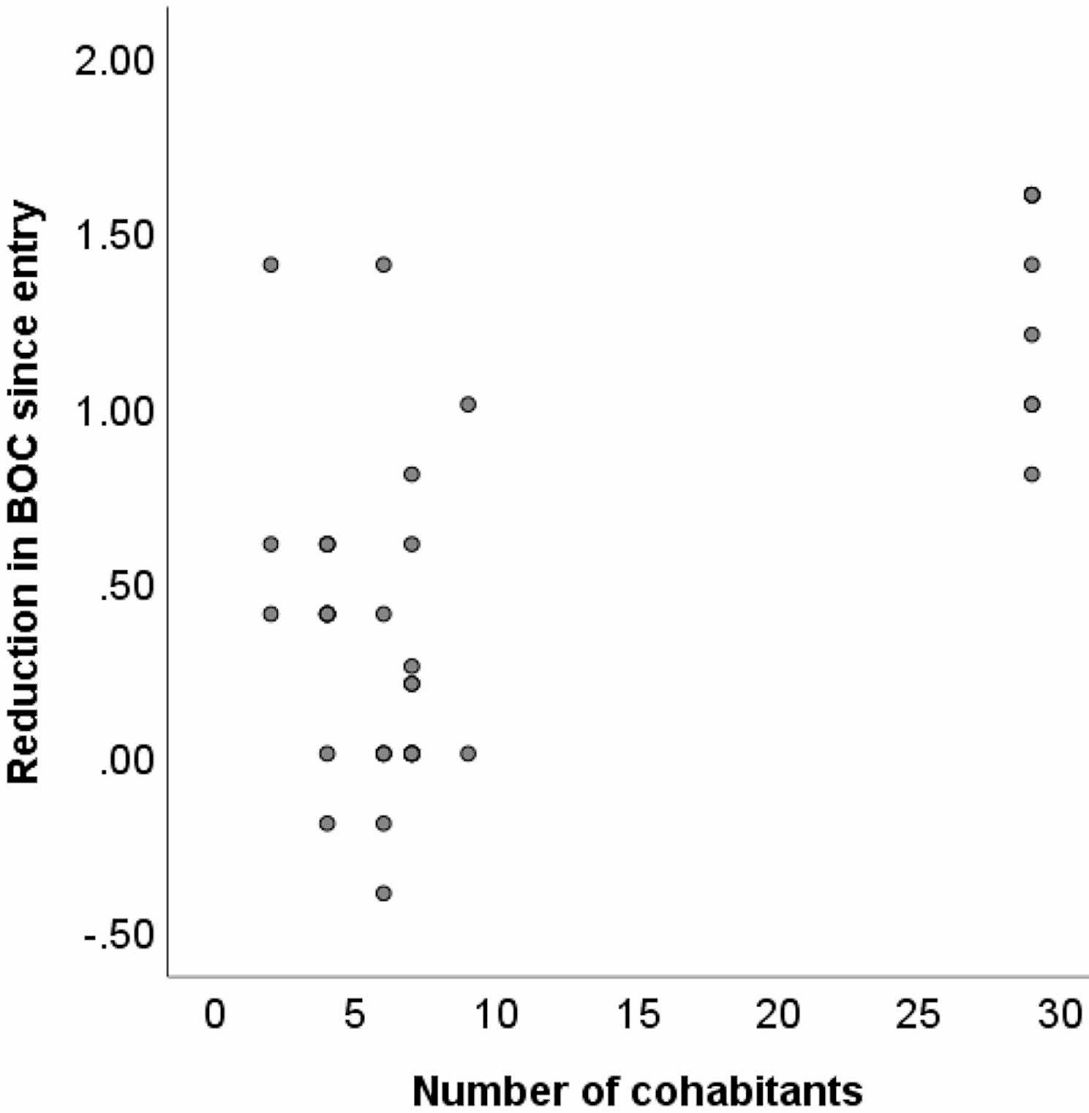
Relationship between changes in behaviours of concern and number of cohabitants Note: Scattergram shows reduction in behaviours of concern (BOC) since entry as a function of number of cohabitants with whom the individual currently lives (*n* = 41)

The role of number of cohabitants in predicting BOC changes over time was interpreted based on visual inspection of a scattergram of the relevant data (Fig. 3). Overall, in the scattergram reflected the significant bivariate correlation reported above, insofar as individuals living with higher the number of cohabitants appear to show greater the reductions in their BOC since entering the service.

Interpretation of this correlation is hampered by the fact that the distribution in number of cohabitants was strongly bimodal, resulting in clustering of outcome variables (Fig. 3). While the majority of individuals lived with fewer than 10 cohabitants, a small number (*n* = 9) lived with almost 30 cohabitants. Cross-referencing with the original dataset revealed that all of these participants lived in the same campus-style residential care setting. Moreover, this subset of individuals was recorded as having shown relatively strong improvements in BOC over time, largely accounting for the overall correlation observed within this scatterplot (Fig. 3). The overall correlation between number of cohabitants and changes in BOC over time remained significant when using non-parametric correlation analysis, which does not require underlying distributions to be normalized (Spearman’s rank correlation coefficient ρ = 0.35, *p* =.023). However, when these nine individuals are removed from the dataset, number of cohabitants is no longer a significant predictor of BOC, whether using either parametric (*r* =.270, *p* =.135) or non-parametric (ρ = 0.28, *p* =.122) correlations.

### Social contact and improvement

**Fig. 4:**
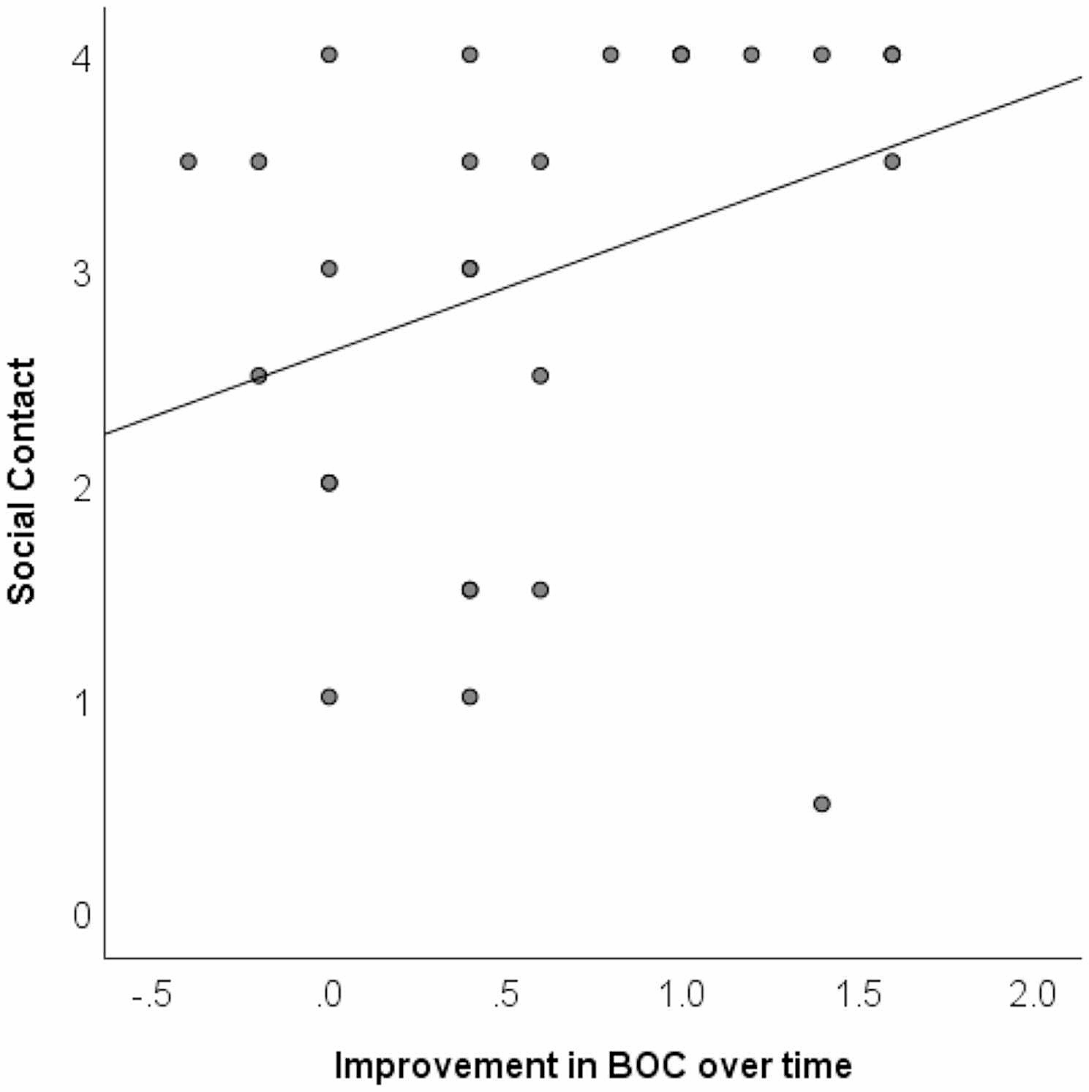
Relationship between social contact and changes in behaviours of concern Note: Scattergram shows current social contact (rated on a scale from 0 to 4) as a function of reduction in behaviours of concern (BOC) since entry (*n* = 29), indicating a significant correlation (ρ = +0.431, *p* =.019)

Information on social contact with family or friends (inside or outside the service context) was available for 36 individuals for whom BMI was also recorded, and for 29 individuals for whom BOC was also recorded. Around a third of individuals had a maximum rating of 4 for overall social contact (family and friends), suggestive of a ceiling effect, and as a result the data were not normally distributed. Current social contact was not significantly correlated with improvements in BMI over time (ρ =–0.248, *p* =.144). However, current social contact was significantly positively correlated with improvements in BOC (ρ = +0.431, *p* =.019) (Fig. 4), indicating that larger improvements in BOC were associated with greater frequency of social contact. While based on a small subset of individuals, this association nonetheless suggests that improvements in individuals’ BOC did not come at the expense of their opportunities for social contact.

## Discussion

Overall, the various analyses reported here, based on accumulated data pooled by a group of 11 full-time care providers from six different countries, suggest that full-time care – i.e., care involving 24-hour staff support typically offered in a residential setting – is associated with improvements in the lives of people who PWS. On average, individuals with PWS were found to experience significant reductions in weight/BMI after joining a full-time specialist care setting, with benefits observable after one year of entering such a setting. These benefits appear to occur regardless of age or initial weight at entry. Similarly, individuals with PWS experienced marked reductions in BOC (temper outbursts, skin-picking, egocentrism, inflexibility, or striving for dominance). Again, these improvements were discernible after one year and were independent of age or severity at entry. These findings support our initial hypothesis that people with PWS who enter care settings gain benefit in terms of reductions in weight and BMI and improvements in behaviours of concern.

The results also suggest that some care settings confer particular benefits that arise from the way services are structured and delivered. Service specialism appears to be especially important. While, on average, individuals living in a variety of residential services experience improvements in weight/BMI, individuals who live in PWS-exclusive services (i.e., who live only with other residents who also have PWS) show the greatest improvements. PWS-exclusive services are likely to operate with a high degree of staff specialisation in PWS care and with strong food control regimens, both of which are also important contributors to reductions in weight/BMI. In mixed service settings, it can be problematic to put appropriate controls on the food environment to meet the needs of service recipients who have PWS, because of the unnecessary restrictions this would create for many other service recipients. Overall, these findings support our second hypothesis that beneficial outcomes are greater in PWS-exclusive rather than in mixed care settings.

PWS specialisation also emerged as an important factor associated with improvements in BOC. Although the relevant data were in short supply, there was some evidence that large service providers – perhaps (we might speculate) those with a high level of accumulated PWS expertise – may be especially effective in supporting behavioural problems in individuals with PWS.

One concern with highly structured care settings is that they might be severely restrictive. While reductions in weight and BOC might reflect improvements in an individual’s overall well-being, the supports required to produce such outcomes may themselves come at a cost: improvements may be secondary to restrictions on an individual’s independence or quality of life. We attempted to probe this matter by examining whether improvements in weight and BOC might come at the expense of social contact with family and friends. In fact, we found the opposite: improvements in BOC were associated with *greater* frequency of social contact. It seems reasonable to interpret this as an illustration of the benefits of reduced BOC: individuals who are supported such that they experience reduced BOC over time are *better able* to engage in social activities as a result. In short, the liberating effects of reduced BOC do not appear to be contingent on restrictions that severely impede an individual’s social life. While our subset of data on social contact and BOC was limited in comparison to the others we scrutinised in this paper, the number of individuals it covered was relatively large when compared to the samples included in most previous studies of full-time PWS care. Overall, these findings support our third hypothesis that improvements in BOC are not associated with restricted social contact.

When considering the implications of these findings, some limitations in the research need to be acknowledged. Chief among these is the fact that the dataset was derived from retrospective archival material gathered independently by service providers for purposes other than empirical research. As such, there will inevitably be inconsistencies in how or when information in the dataset has been recorded. In particular, some records of BOC (for example, records of the intensity of ‘striving for dominance’) may have involved a degree of subjectivity. Furthermore, given the international context of the dataset, some concepts may have been interpreted differently if translated from languages other than English. Overall, future research would benefit from the use of standardised procedures by which primary data can be gathered.

Secondly, the present study is essentially observational in its scope and design. While a number of hypotheses were tested statistically, this was done opportunistically rather than as part of a pre-structured (e.g., experimentally controlled) prospective data collection exercise. That being said, it is important to note that the various hypotheses tested were entirely consistent with an established clinical agenda: namely, the objective of evaluating whether a widely used intervention can be said to be associated with positive (or possibly negative) outcomes. In this regard, it would be difficult to argue that the researchers chose their broad research question arbitrarily or out of convenience. Another consequence of the observational approach was that there was no comparison with a control group, as conclusions were drawn primarily from information relating to individuals with PWS, all of whom lived in full-time care. Nonetheless, while comparisons with a targeted control group would bolster confidence in the interpretation of data, the adverse clinical outcomes seen in people with PWS in the general community are well documented in the medical literature, and so some meaningful inferences can be made. For example, it is notable that participants in the present dataset exhibited marked reductions in body weight, BMI, and BOC, as this is in sharp contrast to the classic trajectory of problems usually encountered by persons with PWS who do not receive appropriate care services.

A third issue to be borne in mind when considering the present findings is the fact that while the dataset is international, it is nonetheless specific in scope. While six different countries were represented, all were high-income Western countries, each ranked by the United Nations Development Programme as being within the top 21 countries in the world for Human Development Index (with four of them ranked inside the top eight). As a result, the present findings are derived from care services located in wealthy societies with well-resourced health and education systems. Any effort to generalise the present results to other global regions will need to take account of some stark variations in economic contexts, legal frameworks, and formal social support infrastructure. Furthermore, we are aware that in some countries, prevailing social norms are for support to always be provided by the (extended) family, with care in residential settings being seen as unacceptable.

A fourth limitation is that the dataset did not contain information on the sex or PWS genotype of the individuals with PWS. Future research should seek to include such data, as well as other potentially informative variables, such as data on sleep, quality of life, medication use, psychiatric diagnoses, and broader health markers. As this was an archival study, the present data were compiled independently of people with PWS themselves, who were not asked to complete any questionnaires or survey instruments. In future studies, information on the perspectives of people with PWS would help elucidate their own reaction to the services they receive, and allow researchers to examine whether the benefits shown above result from extraneous influences (such as the interventions of highly trained support staff), from intrinsic influences (such as improvements in cognitive and social maturity that might develop in the person with PWS), or from a combination of both. While the present study identifies a number of beneficial outcomes, identifying the exact processes that lead to these outcomes will require further research.

Nonetheless, despite these complexities, the findings reported here also benefit from a number of strengths. While it is typical of archival methods to produce limitations on the precision and clarity of data, such limitations are to some extent offset by the relatively large sizes of the datasets that can be collated, allowing researchers greater scope to consider potential caveats and to statistically control for potential confounds. In the present case, the dataset is far larger and richer than any of those used in previous studies of full-time care services for PWS. It covers a range of different age groups, service settings, and nationalities. The size of the dataset allows the testing of multivariate hypotheses with appropriate statistical power, such as the ability to control for age or length of time in a service when examining changes in weight, BMI, and BOC. The size of the dataset also helps to ensure that the observed findings are somewhat representative of a varied cross-section of service experiences and are unlikely to be specific to any single institutional setting, as often is the case with small-scale studies. Overall, the critical mass of data assembled here for analysis highlights the valuable role that collaborative advocacy networks such as IPWSO can play in facilitating the development of evidence-based practice, especially for rare conditions such as PWS.

Another strength of the present study is its consideration of behavioural outcomes as a complement to its evaluation of weight and BMI. Medical assessments of outcomes in PWS frequently focus on weight, and on obesity-related complications, quite possibly because the relevant information is relatively easy to observe and to record. However, personal well-being in PWS is only partly determined by weight or BMI. The quality of life experienced by a person with PWS is very often compromised by a range of other challenges, such as frequent emotional outbursts, domineering behaviour, and skin-picking. While these behaviours can prove complex to define or measure, many service providers do keep person-level records of their frequency and severity. Adding information on BOC to the present dataset allowed for the generation of some useful insights into how full-time care services can be of benefit. In addition, consideration of social contact data helped provide an empirical basis to address concerns about the impact of such services on the broader richness of a person’s life.

## Conclusions

For people with PWS, and for their families, the challenges involved in ensuring that the supports they are provided with are appropriate to meet their highly complex needs can often seem daunting. Moreover, the rarity of the condition severely hampers efforts to produce useful evidence-based treatment recommendations. The findings outlined above serve to highlight the importance of full-time care as a means of supporting people with PWS. In light of our assessment of collaboratively collated international data, we conclude that full-time care services offer people with PWS opportunities to thrive in ways that are both immediate and lasting, especially if those services are specialised around the particular needs that arise from this condition. Individuals with PWS were found to experience significant reductions in weight/BMI after joining a full-time specialist care service, with benefits observable after one year of entering such a service, regardless of age or initial weight at entry. Similarly, individuals with PWS experienced marked reductions in problematic behaviours, such as temper outbursts, skin-picking, egocentrism, inflexibility, or striving for dominance, again discernible after one year and independent of age or severity at entry. The evidence presented above vividly illustrates the potential of full-time professional care to transform the lives of people with PWS, by elevating and maintaining their physical and behavioural well-being.

## Data Availability

The data that support the findings of this study are available from IPWSO (the International Prader-Willi Syndrome Organisation), but restrictions apply to the availability of these data and so they are not publicly available. The data are, however, available from the authors upon reasonable request and with the permission of IPWSO (the International Prader-Willi Syndrome Organisation). [Text based on example from https://www.springernature.com/gp/authors/research-data-policy/data-availability-statements]

## References

[1] Angulo MA, Butler MG, Cataletto ME (2015). Prader-Willi syndrome: a review of clinical, genetic, and endocrine findings. J Endocrinol Invest.

[2] Whittington JE, Holland AJ, Webb T, Butler J, Clarke D, Boer H (2001). Population prevalence and estimated birth incidence and mortality rate for people with prader-Willi syndrome in one UK Health Region. J Med Genet.

[3] Driscoll DJ, Miller JL, Schwartz S, Cassidy SB, Adam MP, Everman DB, Mirzaa GM, Pagon RA, Wallace SE, Bean LJH (2017). Prader-Willi Syndrome. *GeneReviews*®.

[4] Hödebeck-Stuntebeck N. Perspektivwechsel Bei Prader-Willi-Syndrom: Ein Schlüssel Zum Sozialverhalten—Entwicklung Einer Zielgruppenspezifischen Diagnostik und evaluation eines Trainingsprogramms Zur Förderung Der Perspektivübernahme. Logos Verlag; 2012.

[5] Cassidy S, Driscoll D (2009). Prader-Willi syndrome. Pract Genet.

[6] McAllister CJ, Whittington JE, Holland AJ (2011). Development of the eating behaviour in Prader-Willi syndrome: advances in our understanding. Int J Obes (Lond).

[7] Schwartz L, Caixàs A, Dimitropoulos A, Dykens E, Duis J, Einfeld S (2021). Behavioral features in Prader-Willi syndrome (PWS): consensus paper from the International PWS Clinical Trial Consortium. J Neurodev Disord.

[8] Bellis SA, Kuhn I, Adams S, Mullarkey L, Holland A (2022). The consequences of hyperphagia in people with prader-Willi syndrome: a systematic review of studies of morbidity and mortality. Eur J Med Genet.

[9] Miller JL, Tan M (2020). Dietary management for adolescents with prader-Willi syndrome. Adolesc Health Med Ther.

[10] Dykens EM, Roof E, Hunt-Hawkins H (2022). The cure for us is a lot of things’: how young people with prader-Willi syndrome view themselves and future clinical trials. J Appl Res Intellect Disabil.

[11] Kayadjanian N, Schwartz L, Farrar E, Comtois KA, Strong TV (2018). High levels of caregiver burden in Prader-Willi syndrome. PLoS ONE.

[12] Meade C, Martin R, McCrann A, Lyons J, Meehan J, Hoey H (2021). Prader-Willi syndrome in children: quality of life and caregiver burden. Acta Paediatr.

[13] Gallagher L, Roche E, Feighan S-M, Kang H-J, Hughes M. A population-based profile of Prader-Willi syndrome in Ireland. Prader Willi Syndrome Association Ireland; 2017.

[14] Bohonowych J. Living situations for people with PWS change as they get older. *Foundation for Prader-Willi Research Blog*. December 2, 2022. Accessed June 1, 2023. https://www.fpwr.org/blog/living-situations-for-people-with-pws-change-as-they-get-older.

[15] Forster J, ed. *Proceedings of the 2008 and 2009 International PWS Caregivers’ Conferences*: Best Practice Guidelines for Standard of Care in PWS. Absberg H, SoyerNobert Hödebeck-Stuntebeck/IPWSO.; 2010. Accessed December 11, 2023. https://ipwso.org/pws-information/information-for-professional-caregivers/best-practice-guidelines-for-residential-care/.

[16] Mullins JB, Vogl-Maier B (1987). Weight management of youth with prader-Willi syndrome. Int J Eat Disord.

[17] Hirsch HJ, Benarroch F, Genstil L, Pollak Y, Derei D, Forer D (2021). Long-term weight control in adults with prader-Willi syndrome living in residential hostels. Am J Med Genet A.

[18] Bedard KE, Griffith AK, Lister MA, Swain MA (2021). Behavioral and dietary management for adults with prader-Willi syndrome in a residential setting. Adv Neurodev Disord.

[19] Pellikaan K, Rosenberg AGW, Kattentidt-Mouravieva AA, Kersseboom R, Bos-Roubos AG, Veen-Roelofs JMC (2020). Missed diagnoses and health problems in adults with prader-Willi Syndrome: recommendations for screening and treatment. J Clin Endocrinol Metab.

[20] Holland A, Hughes M (2021). Reducing global health inequalities in people with prader-Willi syndrome: the role of the International Prader-Willi Syndrome Organization. Int J Rare Dis Disord.

[21] Mishra P, Pandey CM, Singh U, Gupta A, Sahu C, Keshri A (2019). Descriptive statistics and normality tests for statistical data. Ann Card Anaesth.

[22] McDonald JH (2014). Handbook of Biological statistics.

[23] Cohen J (1988). Statistical Power Analysis for the behavioral sciences.

